# A Pilot Study: N-Staging Assessment of Shear Wave Elastrography in Small Cervical Lymph Nodes for Nasopharyngeal Carcinoma

**DOI:** 10.3389/fonc.2020.00520

**Published:** 2020-04-15

**Authors:** Ying Guan, Shuai Liu, An-Chuan Li, Xin-Bin Pan, Zhong-Guo Liang, Wan-Qin Cheng, Xiao-Dong Zhu

**Affiliations:** ^1^Department of Radiation Oncology, Affiliated Tumor Hospital of Guangxi Medical University, Cancer Institute of Guangxi Zhuang Autonomous Region, Nanning, China; ^2^Department of Radiotherapy Oncology, The Sixth Affiliated Hospital of Sun Yat-Sen University, Guangzhou, China; ^3^Department of Radiation Oncology, Fujian Medical University Union Hospital, Fuzhou, China; ^4^Department of Radiation Oncology, Shunde Hospital, Southern Medical University, Shunde, China

**Keywords:** nasopharyngeal neoplasms, chemoradiotherapy, intensity-modulated radiotherapy, prognosis, shear wave elastrography, cervical lymph nodes

## Abstract

**Purpose:** To investigate N-staging Assessment of pretreatment Shear wave elastrography (SWE) in small cervical lymph nodes (0. 5 cm ≤ maximum diameter < 1 cm, intact capsule, no central necrosis, sCLNs) in nasopharyngeal carcinoma (NPC) patients.

**Methods:** Pathological biopsy proven 28 NPC patients with sCLNs shown in pretreatment magnetic resonance (MR) images and 40 target lymph nodes were enrolled. All target lymph nodes were divided into metastasis and benign lymph node groups according to pathology. SWE was used to exam the real time SWE imaging of each target lymph nodes before conducting ultrasonography guided fine needle biopsy. The minimum (Emin), maximum (Emax), and mean (Emean) elasticity indices (kPa) of target lymph nodes were recorded. The SWE examination was repeated three times for the same target lymph node and each elasticity indices for statistic was determined by average of three measurements. SPSS 21.0 statistics software is used for statistical analysis. The receiver operating characteristic (ROC) curve was performed to obtain the cutoff value of elasticity indices of metastatic sCLNs. Statistical significance was assumed when the *P* < 0.05.

**Results:** Nine lymph nodes were metastatic and 31 were benign. The Emin, Emax, and Emean of benign group were 8.15 ± 6.12, 25.05 ± 12.37, and 16.05 ± 8.29 kPa, respectively; Emin, Emax, and Emean of metastasis group were 11.5 ± 6.17, 41.38 ± 17.87, and 23.48 ± 6.50 kPa, respectively. The difference of the Emax and Emean between metastasis and benign group were statistically significant (*P* = 0.003 and 0.018). The area under the ROC curve of Emin, Emax, and Emean of metastasis lymph node were 0.685 (*P* = 0.095), 0.785 (*P* = 0.010), and 0.765 (*P* = 0.017), respectively. Emax of 27 kPa and Emean of 17 kPa were taken as the cutoff value of diagnosis for metastasis sCLNs: the sensitivity, specificity, and accuracy were 77.8 and 100%, 71.0 and 61.3%, 75.0 and 70.0%, respectively.

**Conclusions:** Pretreatment SWE has high accuracy in evaluating the sCLNs in NPC patients and is helpful for accurate N-staging and survival prognosis. It can be used as a clinical supplementary examination.

## Introduction

N-classification is described a high-risk prognostic factor for distant metastasis and locoregional recurrence in patients with nasopharyngeal carcinoma (NPC). The development of locoregional recurrence has become common pattern of failure in patients who presented with radical radiotherapy. Accurate assessment of cervical lymph node metastases and lymph node benign or metastatic status is critical for evaluating treatment strategies, delineate target volume, delivery radiotherapy, and predicting survival prognosis.

Lymph nodes metastases in NPC follow a predictable and orderly pattern. There was a very low risk of 0.5 to 7.9% in skip nodal metastasis. The two most commonly involved regions at staging were the retropharyngeal and level II lymph nodes. These first echelon nodal groups are followed by Levels III, IV, and V nodal involvement. Levels I and VI nodes have a very low risk for metastasis (1). The distribution of recurrent lymph nodes in NPC is similar to that of initial disease. The recurrence rate in Levels II is high. More than 90% of the recurrence sites are in the original site. The Pattern of lymph nodes metastases in recurrent NPC is similar to that of primary NPC at initial diagnosis. The probability of recurrence in Levels II involvement can be observed to be significantly higher in recurrent NPC. Locoregional recurrence is more than 90% (2).

The diagnostic criteria for cervical lymph node metastases at magnetic resonance (MR) imaging were applied with reference to the criteria proposed by the Chinese Working Committee on Clinical Staging of Nasopharyngeal Carcinoma (CWNPC), which include the following points (3): (a) cervical lymph nodes with a minimal axial diameter of 10 mm or more (level II ≥ 11 mm); (b)lymph nodes of any size with central necrosis or an enhancing rim; (c)three or more lymph nodes in the high-risk area aggregated in a cluster, with a minimal axial diameter of 8 mm or more; and (d)lymph nodes of any size with signs of capsule invasion, including indistinct nodal margins, irregular nodal capsular enhancement, and infiltration into adjacent fat or muscle. The diagnosis of cervical lymph node metastases is identified in accordance with any of the above points. The high-risk area is area orderly following that of the metastatic lymph nodes located.

Although traditional imaging methods have higher sensitivity and specificity than clinically (palpably) positive cervical lymph nodes which meet the diagnostic criteria of MR imaging. In clinical practice, a considerable part of metastatic cervical lymph nodes cannot reach the above diagnostic criteria. Therefore, it is still controversial whether cervical small lymph nodes which do not in accordance with metastatic lymph nodes diagnosis criteria including: the shortest diameter <10 mm and without central necrosis, circular enhancement, and extracapsular invasion should be classified as metastatic lymph node targets in clinical practice target delineation. At present, there are no reliable methods for judging the metastasis of small cervical lymph nodes (maximum short diameter <10 mm without central necrosis, circumferential enhancement, and extracapsular invasion, sCLNs) which do not reach the diagnostic criteria of MR imaging, which makes confusion in delineating the target area of the cervical lymph nodes target for clinical radiotherapist.

Pathological biopsy remains the predominant standard for definitive diagnosis of suspicious cervical lymph node lesions. But because of the particularity of the anatomical structure and pathological type of NPC, cervical pathological biopsy will not be the first choice, and pathological biopsy of each cervical lymph node will not be carried out, so the diagnosis is often made by nasopharyngeal pathological biopsy combined with cervical on palpation or imaging techniques. In clinical practice, the diagnosis of lymphadenopathy basically depends on imaging assessment. For sCLNs < 10 mm, compared with currently available imaging modalities such as ultrasound, computed tomography (CT), and MR imaging, Systemic 2-[F-18] fluoro-2-deoxy-D-glucose positron emission tomography/computed tomography (^18^F-FDG PET/CT) has showed higher promising diagnostic performance (4–6). However, ^18^F-FDG PET/CT also has some limitations in accuracy for assessment sCLNs, such as radioactivity, long examination time, expensive cost, contraindication, and so on. PET/CT is much controversy (7–11), mostly because of the following circumstances: the nodule is small, the infiltration of cancer cells is just beginning, the number of cancer cell clones is small, the uptake of glucose is insufficient or close to the skull base, or the patients' sternocleidomastoid muscle is much strong. The aforementioned circumstances will develop into false-positive or false-negative results. New supplementary diagnostic technologies are still needed to improve the diagnostic accuracy.

Shear wave elastography (SWE) is a novel ultrasound imaging diagnostic imaging technique with quantitative indicators developed in recent years. SWE can be applied to superficial tissues and organs such as thyroid, breast and cervical lymph nodes with high diagnostic efficiency, which can provide a clinical feasibility for differentiating benign from metastatic lesions (12–29). However, few studies have been reported on the diagnostic value for metastatic sCLNs in patients with NPC.

## Materials and Methods

### Patient Selection

The criteria for diagnosing small cervical small lymph nodes (sCLNs) at MR imaging are on T2-weighted images: (a) lymph nodes with a minimal axial diameter of ≥5 and <10 mm in the largest plane; (b) without central necrosis or an enhancing rim; and (c) no signs of extracapsular invasion.

The inclusion criteria were as follows: (a) initial diagnosis of NPC confirmed by biopsy; (b) MR imaging of nasopharynx and cervical was performed for the staging evaluations. Small cervical lymph nodes were seen at MR imaging; (c) no history of prior malignancy; (d) no history of previous anti-cancer treatment; (e) No cervical surgery; (f) No diabetes and other metabolic diseases; and (g) provided written informed consent. The exclusion criteria were those with an axial diameter of <5 mm, difficulties and risk in performing procedure of small cervical lymph nodes adjacent to large blood vessels, or acute inflammation by clinical manifestations.

Newly diagnosed by biopsy-proven NPC patients were eligible, a total of 28 patients was up to the criteria. There were 5 females and 23 males, with a median age of 40 years (range, 19–62 years). MR imaging of nasopharynx and cervical was carried out for the staging evaluations. Six patients also underwent PET/CT at the same time and no radioactivity abnormality was found in sCLNs. According to the 2009, 7th edition of American Joint Cancer Committee on Cancer Staging System Criteria for NPC (7th AJCC), clinical stages demonstrated I, II, III, and IV were 4, 8, 9, and 7, respectively.

### SWE (Shear Wave Elastography) Imaging

**Figure 1:**
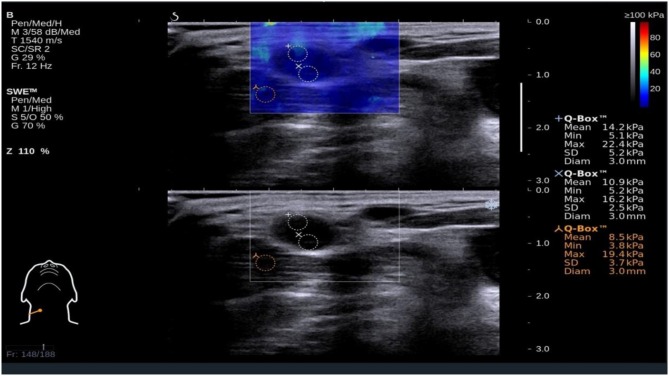
Shear wave elastography evaluation of a benign small cervical lymph nodes (sCLN) biopsy-proven. Shear wave elastography covered the entire target sCLNs and a small amount of surrounding tissue. The shear wave elastography image (up) is presented simultaneously with the gray-scale ultrasound image (below) on the same screen. The node and surrounding tissue exhibited a relatively homogeneous blue color elasticity signal. A 3 mm-diameter Q-box was placed in the node, and the shear elasticity indices of the stiffest region are as follows: Emin (Minimum elasticity values) = 5.2 kPa, Emax (Maximum elasticity values) = 22.4 kPa, and Emean (Mean elasticity values) = 14.2 kPa.

**Figure 2:**
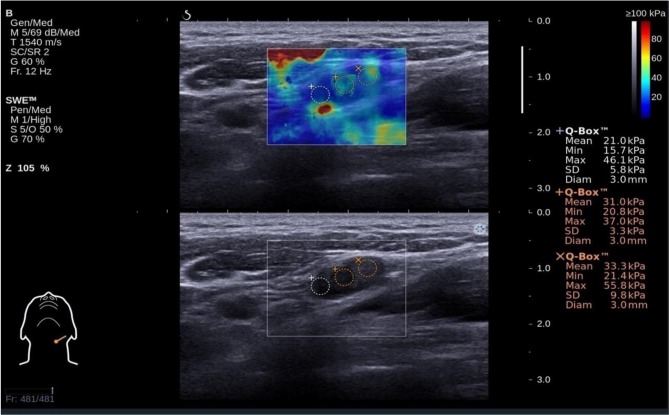
Shear wave elastography evaluation of a metastatic small cervical lymph node (sCLN) biopsy-proven: The shear wave elastography image (up) is presented simultaneously with the gray-scale ultrasound image (below) on the same screen. Shear wave elastography image revealed markedly heterogeneous color between the node lesion and surrounding tissues. The lesion was coded re-yellow. Surrounding tissues were almost pure blue in color. Emin (Minimum elasticity values) = 21.4 kPa, Emax (Maximum elasticity values) = 55.8 kPa, and Emean (Mean elasticity values) = 33.3 kPa.

SWE examinations were performed using a 4–15 MHz transducer linear probe (Supersonic Imagine, Aixen-Provence, France). Before carrying out ultrasono-graphically guided percutaneous biopsy, gray-scale ultrasound examination, and then elastography delivered to every patient. Both ultrasonography and sonoelastography were performed by one radiologist with more than 10 years of experience. During gray-scale ultrasonographic examination, lesion status (length, width, depth, internal echo, and adjacent vascularity) were measured with the aim of identifying the target sCLNs that were observed in the MR images, and elastography followed. The evaluation of sCLNs by SWE was done as described by Desmots et al. (29). During measurement of SWE, a suitable region of interest (ROI; long: 20 mm; wide: 20 mm) covered the gross target sCLNs (a minimal axial diameter of ≥5 mm and <10 mm in the largest plane) and a small part of neighboring tissues. The measurements were taken three times to evaluate the reproducibility. Three Q-boxes (trademark by Supersonic, Imagine, Inc.) 3 mm in diameter (boxes size is predetermined and cannot be changed) were placed on the visibly stiffest region in the lymph node (29). The device automatically generated several elasticity indices (EI) for each Q-box: mean (Emean), minimum (Emin), and maximum (Emax) elasticity values in kPa, as shown in Figures 1, 2. The SWE examination was repeated three times for the same target lymph node because of the relatively small size of Q-box. And each elasticity index for statistic was determined by highest index of three measurements (consideration of nodes incompletely infiltrated by tumor cells). The whole procedure completed in 20–30 min.

### Ultrasound-Guided Core Needle Biopsy (US-CNB)

Total target sCLNs were administrated ultrasound-guided core needle biopsy (US-CNB). Before the US-CNB, we obtained written informed consent for the procedure. The study protocol was approved by our institute review board. The Q-box exhibiting the highest value was regarded as the target area. A 16-gauge core biopsy needle was used for the US-CNB after routine sterilization and local anesthesia (3–5 mL 1% Lidocaine). A free hand approach was used for the CNB procedure with real-time US monitoring. One to three core biopsies of the lymph nodes were performed. The specimens were fixed in 10% formalin and were sent to the pathology department for evaluation. The final diagnosis was decided by tow pathologists with more than 5 years of experience.

### Statistical Analysis

Statistical analyses were conducted using SPSS software version 21.0 (IBM, Armonk, NY). Mean ± standard deviation for quantitate variables. The differences between continuous variables were compared with *t-*test (30, 31). Using the results of pathology diagnosis of puncture biopsy as reference standard, receiver operator characteristic (ROC) curves were constructed for the mean, maximum, and minimum elasticity values. The effectiveness was determined by using the *z*-test. For quantitative variables, the Youden method was used to determine the thresholds of the ROC curves. The values of these parameters maximizing diagnostic accuracy were obtained, and we calculated sensitivity, specificity and accuracy values, and negative (NPV) and positive predictive values (PPV). Statistical significance was assumed when the *P* < 0.05.

## Results

A total of 40 target sCLNs from 28 patients were eligible for inclusion, with a median greatest short axis of 6 mm (range, 5–9 mm) based on the MR imaging. No lymph node capsular invasion or central necrosis was observed. Of all lesions histopathologically evaluated, 31 (~78%) were benign and 9 (~22%) metastatic. Metastatic sCLNs status is listed in Table 1.

**Table 1:** Clinical distribution of metastatic sCLNs status.

**Metastatic sCLNs**	**Initial diagnostic N-staging**	**Size (mm)[Table-fn TN1]**	**Level^**#**^**	**SWE EI (kPa)**			**Ultrasonography**		
				**Emin**	**Emax**	**Emean**	**Hilum of lymph node**	**Blood flow**	**Echo**
1	N0	7	IIa	6.10	52.80	18.50	No	Strip	Homogeneity
2	N0	7	IIa	8.90	56.00	25.10	No	Spot	Homogeneity
3	N2	7	Vb	16.00	27.00	20.40	No	No	Inhomogeneity
4	N0	9	IIb	0.40	71.30	35.50	No	Periphery	Homogeneity
5	N1	6	IIb	21.40	55.80	33.30	No	No	Homogeneity
6	N1	6	IIa	9.60	29.10	20.00	No	No	Inhomogeneity
7	N1	6	IIa	16.10	35.40	20.10	No	No	Inhomogeneity
8	N1	9	IIa	12.50	22.50	19.20	No	Periphery	Homogeneity
9	N1	9	IIb	12.50	22.50	19.20	No	No	Homogeneity

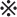
Greatest short axis;

# *According to the consensus guidelines of the lymph node levels; sCLNs, small cervical lymph nodes; EI, Elasticity indices; Emean, Mean elasticity values; Emin, Minimum elasticity values; Emax, Maximum elasticity values*.

According to histopathologic analysis, N-staging was changed in six patients (21.4%), among which two were up-staged from N0 to N1, two from N1 to N2, one from N0 to N2, and one from N2 to N3b.

### Comparison Between Benign and Metastatic sCLNs

Color Doppler ultrasound-graphic evaluation of all target sCLNs prior to performance of biopsy: it was no significant difference between benign and metastatic sCLNs groups. Twelve lymph nodes (30%) had hilum of lymph nodes, 28 lymph nodes (70%) were no hilum of lymph nodes; 21 lymph nodes (52.5%) showed no blood flow, 19 lymph nodes (47.5%) showed blood flow (22.5% peripheral blood flow, 17.5% spot shape blood flow, 5% portal shape blood flow; 2.5% were strip shape blood flow); internal echo of 33 lymph nodes (82.5%) were homogeneity and 7 lymph nodes (17.5%) were inhomogeneity (Table 2).

**Table 2:** Statistical analysis of color doppler ultrasound-graphic evaluation between benign and metastatic sCLNs groups.

	**Benign sCLNs** **(*n* = 31)**	**Metastatic sCLNs** **(*n* = 9)**	**χ^2^**	***P***
Hilum of lymph nodes			2.422	0.120
Yes/No	12/19	0/9		
Blood flow shape			4.317	0.365
No	16	5		
Periphery/Spot/Port/Strip	7/6/2/0	2/1/0/1		
Internal echo homogeneity			2.016	0.156
Yes/No	27/4	6/3		

*sCLNs, small cervical lymph nodes*.

**Figure 3:**
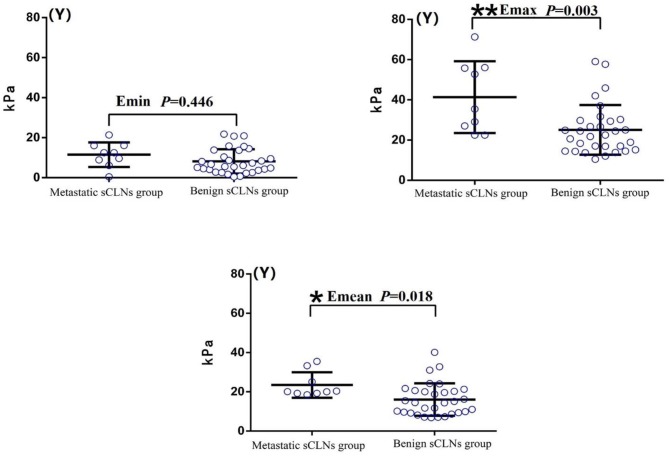
Comparison of elasticity values (*Y*-axis) of Shear wave elastography between benign and metastatic small cervical lymph nodes (sCLNs) groups in scatter plot. There was no significant difference between the Emin (Minimum elasticity values) between benign and metastatic sCLNs groups (*P* = 0.446 > 0.05). The differences of the Emax (Maximum elasticity values) and Emean (Mean elasticity values) between benign and metastatic sCLNs groups were statistically significant (**P = 0.003 < 0.005 and *P = 0.018 < 0.05).

Shear wave elastography evaluation of all target sCLNs: There was no significant difference between the Emin between benign and metastatic sCLNs groups (*P* = 0.446 > 0.05). The differences of the Emax and Emean between benign and metastatic sCLNs groups were statistically significant (*P* = 0.003 < 0.005 and *P* = 0.018 < 0.05; Figure 3). The Emin, Emax, and Emean of benign sCLNs group were 8.15 ± 6.12, 25.05 ± 12.37, and 16.05 ± 8.29 kPa, respectively; Emin, Emax, and Emean of metastatic sCLNs group were 11.5 ± 6.17, 41.38 ± 17.87, and 23.48 ± 6.50 kPa, respectively (Table 3).

**Table 3:** Statistical analysis of SWE between benign and metastatic sCLNs groups.

**EI**	**Benign sCLNs** **(*n* = 31)**	**Metastatic sCLNs** **(*n* = 9)**	***t***	***P***
Emin (kPa)	8.15 ± 6.12	11.5 ± 6.17	−0.79	0.446
Emax (kPa)	25.05 ± 12.37	41.38 ± 17.87	−4.50	0.003
Emean (kPa)	16.05 ± 8.29	23.48 ± 6.50	−2.65	0.018

*SWE, Shear wave elastography; sCLNs, small cervical lymph nodes; EI, Elasticity indices; Emean, Mean elasticity values; Emin, Minimum elasticity values; Emax, Maximum elasticity values*.

### Diagnostic Performance of SWE

**Figure 4:**
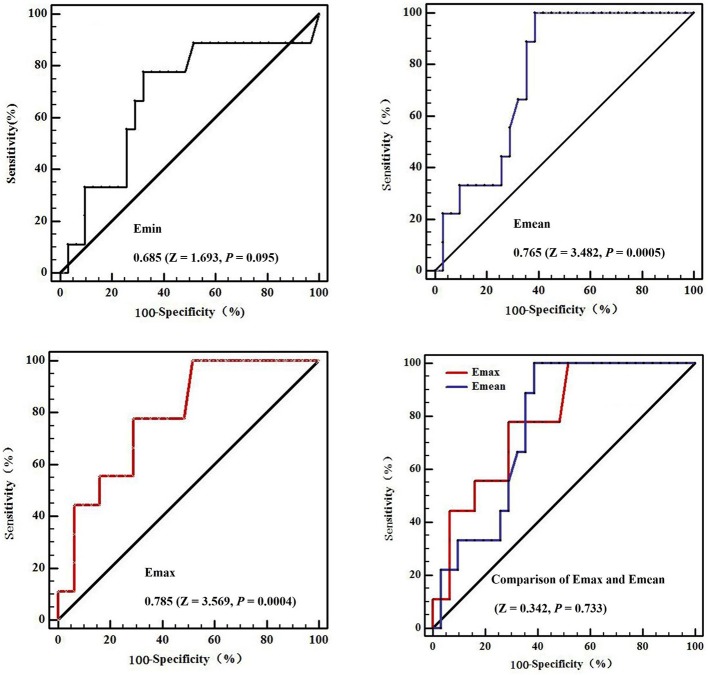
The receiver operating curves (ROC) in diagnostic performance of Shear wave elastography for metastatic small cervical lymph nodes. The areas under the ROC curves were 0.685 (*Z* = 1.693, *P* = 0.095) for Emin (Minimum elasticity values) = 0.785 (*Z* = 3.569, *P* = 0.0004) for Emax (Maximum elasticity values), and 0.765 (*Z* = 3.482, *P* = 0.0005) for Emean (Mean elasticity values). Further *Z*-test compared the areas under Emax and Emean ROC curves, there was no significant difference between them, and their diagnostic performance was similar (*Z* = 0.342, *P* = 0.733).

The areas under the ROC curves were 0.685 (*Z* = 1.693, *P* = 0.095) for minimum elasticity (Emin), 0.785 (*Z* = 3.569, *P* = 0.0004) for maximum elasticity (Emax), and 0.765 (*Z* = 3.482, *P* = 0.0005) for mean elasticity (Emean). Further *Z*-test compared the areas under Emax and Emean ROC curves, there was no significant difference between them, and their diagnostic performance was similar (*Z* = 0.342, *P* = 0.733; Figure 4). The cutoff values affording the maximal predictive diagnosis were 27 and 17 kPa, respectively for the Emax and Emean. The accuracy, sensitivities, specificities, NPVs, and PPVs derived using these values are summarized in Table 4.

**Table 4:** Diagnostic performances of SWE for metastatic sCLNs in NPC patients.

**EI**	**Cutoff values** **(kPa)**	**Sensitivity** **(%)**	**Specificity** **(%)**	**PPV** **(%)**	**NPV** **(%)**	**Accuracy** **(%)**
Emax	27	77.8	71.0	43.8	91.6	72.5
Emean	17	100	61.3	42.9	100	70.0

*SWE, Shear wave elastography; sCLNs, Small cervical lymph nodes; NPC, Nasopharyngeal carcinoma; EI, Elasticity indices; Emean, Mean elasticity values; Emin, Minimum elasticity values; Emax, Maximum elasticity values; PPV, Positive predictive values; NPV, Negative predictive values*.

## Discussion

In the study, the Emin, Emax, and Emean of benign sCLNs group were 8.15 ± 6.12, 25.05 ± 12.37, and 16.05 ± 8.29 kPa, respectively; Emin, Emax, and Emean of metastatic sCLNs group were 11.5 ± 6.17, 41.38 ± 17.87, and 23.48 ± 6.50 kPa respectively. This is similar to previous SWE studies showing that the elasticity indices of metastatic cervical lymph nodes are higher than that of benign cervical lymph nodes.

There are about 800 lymph nodes in the human body, including about 300 lymph nodes in the neck (32). Accurate evaluation of the presence of metastatic cervical lymph nodes is the most important factor for the prognosis and planning of treatment in head and neck tumors. Regardless of the site of the primary tumor, the presence of a single metastatic lymph node in either the ipsilateral or contralateral side of the neck reduces the 5 year survival rate by about 50%. The presence of a single metastatic lymph node in each side of the neck reduces the survival rate to nearly 25% of that expected in patients without any cervical lymph nodal metastasis (33).

The relationship between lymph node size and cancer metastasis: (a) There is a certain correlation between the two: large lymph nodes have high detection incidence of tumor deposits; (b) But the relationship between the two is not absolutely: large lymph nodes may also could be benign. Regional lymph nodes metastasis is a predominant feature of head and neck squamous cell carcinoma (HNSCC) and is also a significant survival prognostic factor. Clinically N0 neck (cN0) for tumors with significant (≥20%) incidence of occult regional metastasis (34). The challenge is to evaluate the absence/presence of lymph node metastases in scattered sCLNs on pretreatment imagings. It is one of the most important prognostic factors for radiotherapists and radiologists in evaluating the severity and the prognosis of disease. At the same time, when doctors making clinical treatment plans, these metastatic sCLNs which do not meet the diagnostic criteria don't receive radical radiotherapy which was a main reason for lymph nodes recurrence after radiotherapy. Although imaging has improved in the last decades, it is a limitation common to all current imaging techniques which lack of sensitivity for little tumor deposits (34).

SWE plays an important complementary role in distinguishing benign and metastatic lymph nodes. We found data of published SWE studies (26–29) on cervical lymph nodes in Table 5: (a) Emax or Emean were basically selected as the metastatic lymph nodes diagnostic cutoff values, mostly above 10 kPa; (b) the elasticity indices of metastatic lymph nodes were higher than that of benign lymph nodes.

**Table 5:** The published SWE studies on sCLNs.

**Study**	**Pathology**	**Cutoff** **(kPa)**	**EI** **(kPa)**	**Sensitivity (%)**	**Specificity (%)**	**Accuracy (%)**
Bhatia et al. (26)	SCC (11), Lung adenocarcinoma (5), NHL (4), NPC (5), PTC (5), Poorly differentiated carcer (1)	Emean = 30.2	Benign: 21.4 and Metastatic: 25	41.9	100	61.8
Choi et al. (27)	PTC (26), Salivary gland carcinoma (1), Hypopharynx SCC (6), Melanoma (1)	Emax = 19.44	Benign: Emax = 14.22 ± 4.19 and Metastatic: Emax = 41.06 ± 36.34	91	97	94
Jung et al. (28)	PTC (51)	Emean = 29	Benign: Emin = 18.70 ± 11.82, Emax = 35.52 ± 29.16, Emean = 27.50 ± 18.95 and Metastatic: Emin = 48.49 ± 47.21, Emax = 79.61 ± 71.23, Emean = 67.93 ± 62.52	98	45.4	77.38
Desmots et al. (29)	Squamous carcinomas (16), papillary thyroid carcinomas (4), head and neck (2), sarcomas (2), lymphomas (2), kidney (2), melanoma (1), malignant paraganglioma (1) Reference standard: pathology	Emax = 31.0	Benign: Emax = 23.3 ± 25.3 and Metastatic: Emax = 72.4 ± 59.0	86.7	87.5	87.1

*SWE, Shear wave elastography; sCLNs, Small cervical lymph nodes; NPC, Nasopharyngeal carcinoma; SCC, Squamous cell carcinomas; PTC, Papillary thyroid carcinomas; NHL, Non-Hodgkin lymphoma; EI, Elasticity indices; Emean, Mean elasticity values; Emin, Minimum elasticity values; Emax, Maximum elasticity values*.

In current study, pathological diagnosis after US–CNB was used as the unique standard. Emax of 27 kPa (*P* = 0.0004) and Emean of 17 kPa (*P* = 0.0005) were taken as the cutoff point of diagnosis for metastatic sCLNs: the sensitivity, specificity, and accuracy were 77.8 and 100%, 71.0 and 61.3%, 75.0 and 70.0%, respectively. The diagnostic elasticity indices, sensitivity, specificity, and accuracy of differentiating benign and metastatic sCLNs shown in this study are different from those of the above-mentioned studies (summarized in Table 5). Consideration may be due to the following reasons: (a). Previous studies were all cervical lymph nodes that reach the diagnostic criteria by clinical examination or by imaging, but none of sCLNs meet the clinical diagnostic criteria in this study. The development of cervical lymph node metastasis is a dynamic continuous procedure: in the early stage of metastasis, a small tumor cells deposit implanted into the marginal sinus of lymph nodes through lymphatic vessels, and then into the medullary sinus. A large number of cloned tumor cells filled the whole lymph node, which significantly increased the density of tumor cells in lymph nodes, increased the tension, and lymphadenopathy. At the end of this procedure, when the lymph node is completely infiltrated by tumor cells, the tumor cells break through the envelope and adhere to the surrounding vascular tissue, accompanied by interstitial fibrous tissue proliferation, resulting in lymph node fixation and further development in stiffness and elasticity indices. Therefore, the elasticity indices of cervical lymph nodes are different in different stages and extents of involvement; (b). In this study, we focus on scattered sCLNs in the neck of NPC patients. There were many various tumors pathological types in previous studies. Also, the study of thyroid and breast cancer also confirmed that different pathology, lesions size, extracapsular invasion, lymphatic vessel infiltration, and pathology diagnosis methods can affect the elasticity indices (14, 17, 18, 20, 21).

For head and neck malignant tumors, the accurate rate of US–CNB was 93%. Because of the “seed soil hypothesis” (35), both seed and soil factors play a pivotal role in mediating metastasis. The seed depends on supportive soil; however, it is important to note the dynamic interplay between seed and soil affect each other and evolve together. Therefore, the heterogeneity of tumor cells infiltration in lymph nodes is not uniform, and the limited sample size makes it difficult for even experienced pathologists to distinguish between benign and metastatic lesions.

## Conclusion

Pretreatment SWE is a promising reproducible quantitative tool with which to predict metastasis especially sub-centimeter nodes-sCLNs (lymph nodes with a minimal axial diameter of ≥5 and <10 mm in the largest plane; without central necrosis or an enhancing rim and no signs of extracapsular invasion) for NPC patients and is helpful to N-staging and survival prognosis. It can be used as a clinical supplementary examination for traditional imaging examinations such as MRI and CT. A prospective study is approved to affirm the results we gained from our retrospective analysis and we have already started to study on it.

## Data Availability Statement

All datasets generated for this study are included in the article/supplementary material.

## Ethics Statement

The studies involving human participants were reviewed and approved by Affiliated Cancer Hospital of Guangxi Medical University review board. The patients/participants provided their written informed consent to participate in this study. Written informed consent was obtained from the individual(s) for the publication of any potentially identifiable images or data included in this article.

## Author Contributions

YG, SL, and A-CL contributed with study design, data analysis, interpretation of findings, and writing of the manuscript. X-BP and Z-GL contributed with data collection. W-QC and X-DZ contributed with literature research. All authors read and approved the final manuscript.

### Conflict of Interest

The authors declare that the research was conducted in the absence of any commercial or financial relationships that could be construed as a potential conflict of interest.
